# The influence of mother’s embrace on the level of infant pain during injection

**DOI:** 10.1186/1471-2458-12-S2-A35

**Published:** 2012-11-27

**Authors:** Arie Kusumaningrum, Regina Natalia

**Affiliations:** 1 School of Nursing, Faculty of Medicine, Sriwijaya University, Indralaya, Indonesia

## Background

Administering injections on infants, as one of routine parenteral procedures can cause pain to the infant. A mother’s embrace while the infant is being given an injection may provide pain relief, as a form of non-pharmacological intervention. This research was conducted to determine the influence of mothers’ embrace on the level of infant pain during an injection.

## Materials and methods

This study was a quasi-experimental design involving one intervention group and one control group which were not subjected to randomization. Study participants were 24 infants and their mothers (14 control, 10 intervention) who were recruited upon attendance for routine vaccination at Indralaya Public Health Centre. Participants were selectedusing convenient sampling. The size of needle, location and type of injection were controlled to ensure standardization of vaccination methods.

## Results

Infants in this study were aged 177,71 ± 80,98 days (mean ±SD) and weighed 091,67 ± 1789,32 gram. They received injections for the following immunizations: DPT (55%), Measles (29.3%) and Combo (15.7%). Levels of pain as measured using FLACC (Face, leg, activity, cry and consolability pain index) and FACE (Wong-Baker pain scale) parameter were significantly lower for the intervention group compared to the control (P < 0.001, Mann Whitney test). FLACC and duration of FACE parameters were not affected by age of infants, weight of infants, and immunization type.

## Conclusions

Being held in their mother’s embrace while being given an injection can significantly reduce the level of pain felt by an infant. Hence, it is recommended for health workers to apply this intervention as a non-pharmacological management to reduce infant pain during injection.

